# Production and *N*-glycan engineering of Varlilumab in *Nicotiana benthamiana*

**DOI:** 10.3389/fpls.2023.1215580

**Published:** 2023-08-08

**Authors:** Kim Dua Nguyen, Hiroyuki Kajiura, Ryo Kamiya, Takahiro Yoshida, Ryo Misaki, Kazuhito Fujiyama

**Affiliations:** ^1^ International Center for Biotechnology, Osaka University, Osaka, Japan; ^2^ Industrial Biotechnology Initiative Division, Institute for Open and Transdisciplinary Research Initiatives (OTRI), Osaka University, Osaka, Japan; ^3^ GreenLand-Kidaya Group Co Ltd., Fukui, Japan; ^4^ Osaka University Cooperative Research Station in Southeast Asia (OU: CRS), Faculty of Science, Mahidol University, Bangkok, Thailand

**Keywords:** cancer immunotherapy, galactosyltransferase, hydroponics, monoclonal antibody (mAb), *Nicotiana benthamiana*, Varlilumab

## Abstract

*N*-glycan engineering has dramatically evolved for the development and quality control of recombinant antibodies. Fc region of IgG contains two *N*-glycans whose galactose terminals on Fc-glycan have been shown to increase the stability of CH2 domain and improve effector functions. *Nicotiana benthamiana* has become one of the most attractive production systems for therapeutic antibodies. In this study, Varlilumab, a CD27-targeting monoclonal antibody, was transiently produced in fresh leaves of soil-grown and hydroponic-grown *N. benthamiana*, resulted in the yield of 174 and 618 µg/gram, respectively. However, the IgG produced in wild-type *N. benthamiana* lacked the terminal galactose residues in its *N*-glycan. Therefore, *N*-glycan engineering was applied to fine-tune recombinant antibodies produced in plant platforms. We further co-expressed IgG together with murine β1,4-galactosyltransferase (β1,4-GALT) to modify plant *N*-glycan with β1,4-linked Gal residue(s) and *Arabidopsis thaliana* β1,3-galactosylatransferase (β1,3-GALT) to improve galactosylation. The co-expression of IgG with each of GALTs successfully resulted in modification of *N*-glycan structures on the plant-produced IgG. Notably, IgG co-expressed with murine β1,4-GALT in soil-grown *N. benthamiana* had 42.5% of *N*-glycans variants having galactose (Gal) residues at the non-reducing terminus and 55.3% of that in hydroponic-grown *N. benthamiana* plants. Concomitantly, *N-*glycan profile analysis of IgG co-expressed with β1,3-GALT demonstrated that there was an increased efficiency of galactosylation and an enhancement in the formation of Lewis a structure in plant-derived antibodies. Taken together, our findings show that the first plant-derived Varlilumab was successfully produced with biantennary β1,4-galactosylated *N*-glycan structures.

## Introduction

The use of therapeutic monoclonal antibodies for the treatment of cancers, Alzheimer’s disease, and other conditions (Perneczky et al., 2023) has increased substantially over the past years (Kaplon et al., 2023). Accordingly, there has been a demand for high-yield production of recombinant antibodies at low cost. Recently, whole-plant *Nicotiana benthamiana* was selected as a host for such recombinant protein production due to its various advantages, including low production cost, absence of animal contamination, high biomass, and fast and easy scale-up (Sheshukova et al., 2016). These characteristics make *N. benthamiana* an effective alternative production platform for biopharmaceuticals. Moreover, *N. benthamiana* is efficient for agroinfiltration-based transient expression of a variety of antibodies (Sainsbury and Lomonossoff, 2008; Grohs et al., 2010). For instance, ZMapp, a cocktail of three plant-derived antibodies for neutralizing Ebola virus (Qiu et al., 2014), and Covifenz (Medicago), the first plant-based virus-like particles for vaccine against SARS-COV-2 (Pillet et al., 2022), have demonstrated the advantages of *N. benthamiana* over other production platforms during the pandemics. In addition, one of the plant-growing systems is the hydroponic system, which is a method of growing plants in nutrient solutions with or without the use of a mechanical-support medium. The solutions in this system are circulated by a pump, which allows nutrient recovery and minimizes water loss in sustainable agriculture (Kumar and Cho, 2014). Hydroponic system is closed and free from soil-borne diseases, so it was chosen to produce MucoRice-CTB (a rice-based oral vaccine against cholera) to qualify the current good manufacturing practice (cGMP) regulations (Kashima et al., 2016). Several hydroponic systems for *Arabidopsis* and other model plants have been developed (Alatorre-Cobos et al., 2014). Recently, hydroponic system was also employed to transiently produce two SARS-CoV-2 neutralizing antibodies in *N. benthamiana* modified plants (Frigerio et al., 2022). Therefore, the merit of hydroponic-based *N. benthamiana* in producing recombinant proteins such as antibodies should be extensively investigated.

Varlilumab is a human monoclonal antibody IgG1 that targets CD27, a receptor belonging to the tumor necrosis factor (TNF) receptor superfamily. CD27 is well known for its important role in T-cell immunity and as a cell-surface marker of B- and T-cell malignancies. Varlilumab was initially generated using Ig transgenic mice and developed as a potential therapeutic agent for CD27-expressing lymphoma and leukemia (Vitale et al., 2012). In a later study, Varlilumab exhibited potent antitumor activity as both monotherapy and combination therapy in preclinical models (Wasiuk et al., 2017). In combination with Nivolumab (a PD-1 immune checkpoint inhibitor antibody), Varlilumab has shown success in a phase 2 clinical trial as an anti-CD27 agonist antibody against recurrent glioblastoma (Reardon et al., 2018). The safety and activity of Varlilumab have also been evaluated in patients with advanced solid tumors (Burris et al., 2017).

Varlilumab and other IgG molecules contain an *N*-glycosylation site at Asn297 of the Fc region, which plays a critical role in Fc effector functions, such as antibody-dependent cellular cytotoxicity (ADCC) and complement-dependent cytotoxicity (CDC) (Mimura et al., 2018). As a result, *N*-glycan engineering of the Fc regions is a rational strategy for improving the safety and efficacy of recombinant antibodies. *N*-glycosylation occurs first in the endoplasmic reticulum and is subsequently processed during the transport through Golgi apparatus compartments (Seemann et al., 2000; Bard et al., 2006). At each compartment, there are varying compositions of *N*-glycosylation enzymes which catalyze *N*-glycan extension reactions on the secretory *N*-glycoproteins (Elsner et al., 2003). *N*-glycosylation facilitates the correct folding of proteins and provides protease resistance and solubility (Varki, 2017). As mentioned earlier, antibodies are *N*-glycoproteins carrying conserved *N*-glycans at the Fc domain. Different *N*-glycosylation patterns can profoundly affect the biological and therapeutic functions of antibodies (Wang et al., 2019). Highly β1,4-galactosylated antibodies exhibit higher affinity for FcγRIIIa and greater thermal stability because the galactose moiety decreases the conformational entropy of CH2, facilitating the engagement of Fc to FcγRIIIa. Thus, β1,4-galactosylated antibodies possess higher ADCC activity and CDC activity (Kiyoshi et al., 2018). β1,4-galactosyltransferase (β1,4-GALT) catalyzes the transfer of galactose (Gal) from UDP-Gal to the *N*-acetylglucosamine (GlcNAc) residue at the non-reducing terminal of *N*-glycan in mammals, whereas *N. benthamiana* plants lack this enzyme in the Golgi apparatus and instead have β1,3-galactosyltransferase (β1,3-GALT).

Previous studies have focused on expressing human β1,4-GALT in transgenic tobacco cell lines (Palacpac et al., 1999) and transgenic plants of *N. tabacum* (Bakker et al., 2001). In *N. benthaminana*, substantial efforts have been made to achieve plant-derived recombinant proteins with galactose terminated *N*-glycan structures. Notably, different variants of human β1,4-GALT were introduced in *N. benthamiana* to increase the level of biantennary galactosylated *N*-glycans (Hesselink et al., 2014) and multigene vectors containing human sialylation pathway including β1,4-galactosylation were transformed into *N. benthamiana* plants for the generation of complex sialylated structures (Kallolimath et al., 2016). However, these studies employed stable expression in plant cell-lines or transgenic plants, which is a slow and labor-intensive approach (Rivera et al., 2012; Sethi et al., 2021). To circumvent this bottleneck, transient co-expression of β1,4-GALT in *N. benthamiana* was studied (Vézina et al., 2009). This strategy realized a high yield and allowed the fast modification of *N*-glycan profiles of recombinant antibodies. Still, the expression of the original β1,4-GALT or a fusion of β1,4-GALT and N-acetylglucosaminyltransferaseI (GNTI) construct might block the action of other enzymes such as Golgi-α-mannosidase II, which would also result in partially processed glycans (Saint-Jore-Dupas et al., 2006; Vézina et al., 2009). Significant efforts have been contributed to improve β1,4-galactosylation of IgG by targeting β1,4-GALT to the *trans*-Golgi compartments in both stable and transient expression of *N. benthamiana* (Strasser et al., 2009; Forthal et al., 2010; Castilho et al., 2011; Loos et al., 2015; Schneider et al., 2015; Kallolimath et al., 2018). One of the impressive achievements was the transient co-expression of ^ST^GalT binary vector with IgG in a glyco-engineered ΔXTFT *N. benthamiana* plants. ^ST^GalT is a chimeric protein consisting of cytoplasmic tail, transmembrane domain and stem (CTS) region of α2,6-sialyltransferas (ST). This co-expression obtained more than 60% of the structures carried galactose terminals after 4 days post-infiltration (Castilho et al., 2011). It is suggested that these approaches were effective to achieve correct localization of β1,4-GALT and hybrid *N*-glycan structures in plants. In previous studies, the CTS of mammalian α2,6-ST was chosen for the fusion with the catalytic domain of β1,4-GALT because this enzyme targeted to the *trans*-Golgi compartments and successfully modified the *N*-glycan profile of plant-derived IgGs. To further investigate and establish simple *in vivo N*-glycan modification steps for the generation of biantennary galactosylated IgG in transient expression of *N. benthamiana*, β1,3-GALT was chosen because the subcellular localization of β1,3-GALT was predicted to be exclusively located in the plant Golgi apparatus. Unlike mammalian β1,4-GALT, plant β1,3-GALT transfers a Gal to a terminal GlcNAc residue in β1,3-linkage (Strasser et al., 2007). This suggests that both mammalian β1,4-GALT and plant β1,3-GALT may have similar localization patterns.

In this study, we expressed recombinant Varlilumab in soil- and hydroponic-based *N. benthamiana* plants. The *N*-glycan profiles of plant-derived Varlilumab were independently modified by introducing β1,4-GALT and over-expressing β1,3-GALT. Our work demonstrated that the first plant-derived Varlilumab was successfully produced with biantennary β1,4-galactosylated *N*-glycan structures using a chimeric β1,3β1,4-GALT.

## Materials and methods

### Plant expression vector construction

Genes encoding variable regions of Varlilumab H and L chains (clone 1F5, US 2011/0274685 A1) were synthesized by Invitrogen (Carlsbad, CA; USA). The coding regions of H and L chains were introduced to pQCXIP (TaKaRa Bio, Otsu, Japan) and pQCXIH (TaKaRa Bio) while retaining the human H and L, respectively, to form the full-length Varlilumab H and L chains. The modified pPK1-BAR binary plasmid was previously constructed (Sariyatun et al., 2021). Inserts of full-length H and L chains were amplified from pQCXIP-HC and pQCXIH-LC with the addition of *Xba* I sites, respectively. VarHC-*Xba*I-Fw 5’-TAC TCT AGA ATG GAG TTT GGG CTG AGC TGG G-3’ and VarHC-*Xba*I-Rv 5’-TAC TCT AGA CTA TTT ACC CGG AGA CAG GGA GA-3’ were used for the amplification of Varlilumab H, while Varlilumab L was amplified by the primer pair VarLC-*Xba*I-Fw 5’ TAC TCT AGA ATG AGG GTC CTC GCT CAG CT-3’ and VarLC-*Xba*I-Rv 5’- TAC TCT AGA CTA ACA CTC TCC CCT GTT GAA GCT-3’ (the *Xba* I restriction site is underlined). pFK1-BAR fragments were digested by *Spe*I and the inserts were cleaved by the *Xba* I restriction enzyme (New England Biolabs, Beverly, MA) and subsequently ligated using Ligation High Ver. 2.0 (Toyobo, Osaka, Japan). Both *Sp*e I and *Xba* I produced compatible sticky ends (CTAG), so the digested products can be ligated. This resulted in circular binary plasmids containing H and L chain-expression cassettes, which were designated pFK1-BAR-VarHC and pFK1-BAR-VarLC. cDNAs encoding for *A. thaliana* β1,3-GALT (At1g26810) and murine β1,4-GALT were isolated and then introduced in the *Nde* I/*Sac* I restriction site of the binary plasmid pRI201-AN (TaKaRa Bio).

### Agroinfiltration

The vectors expressing the Varlilumab H and L chains and GALTs were inserted into *Agrobacterium tumefaciens* strain LBA4404 by electroporation (Bio-Rad, Hercules, CA) with voltage, resistance, and capacitance at 2.4 kV, 200 Ω, and 25 µF, respectively. The RNA silencing suppressor 19 (p19) vector was kindly provided by Prof. Atsushi Takeda (Ritsumeikan University). *A. tumefaciens* transformants were selected using a combination of kanamycin (50 mg/L), rifampicin (50 mg/L), and streptomycin (50 mg/L). A single colony of *Agrobacterium* was first inoculated into 5 ml of 2xYT liquid medium with the above-mentioned antibiotics and cultivated at 28°C overnight, followed by a continuous cultivation of 200 ml of the thus-prepared medium. Cells were collected by centrifugation at 4,000 x *g*. *Agrobacterium* harboring different vectors (H, L, RNA silencing p19 or GALTs) was resuspended and mixed in modified infiltration buffer (10 mM MgSO_4_, 10 mM MES, 0.56 mM ascorbic acid, 0.03% Tween-20, 100 mM acetosyringone, pH 5.8) at OD_600_ 0.5 following the method of Zhao et al. (2017). The *Agrobacterium* containing H or L was mixed at a 1:1 ratio for expression of Varlilumab. For co-expression with the RNA silencing suppressor p19, the ratio was 1:1:2 for H, L, and p19, respectively. In GALT co-expression experiments, the ratio was 1:1:1:1 for four different constructs (H, L, p19 and GALT). *N. benthamiana* plants were infiltrated by vacuum infiltration (Chen et al., 2013). Infiltrated plants were grown in a controlled plant room with a 16 h light/8 h dark cycle at 28°C for 10 days. The hydroponic-based *N. benthamiana* wild type plants used in this study were prepared by the GreenLand-Kidaya Group (Fukui, Japan). Briefly, *N. benthamiana* seeds were sown into a urethan board in the dark at 21°C. Then, the germinated seedlings were grown at 21°C under a 16 h LED-photoperiod of 420 mmol/m^2^s (Yumex Co., Hyogo, Japan) for 10 days, followed by a 16 h yellow LED-photoperiod of 220 mmol/m^2^s (RAYS Co.) for 20 days. The nutrient solution used was a mixture of OAT House 1 (71%), OAT House 8 (26%) and OAT House 5 (3%) (all from OAT Agrio Co., Tokyo, Japan). The system was washed with detergent and rinsed twice with tap water between two independent experiments.

### Protein expression, SDS-PAGE, and western blotting analysis

Infiltrated leaves were harvested on 2, 4, 6, 8 and 10 days post-infiltration (dpi) to evaluate the transient expression level of antibody. Leaf samples were homogenized in liquid nitrogen using a mortar and pestle, followed by protein extraction in 100 mM sodium phosphate, 100 mM sodium chloride and 40 mM ascorbic acid at pH 6.0 (Husk et al., 2014). The CHO-derived human Varlilumab (Thermo Fisher Scientific, Waltham, MA) and plant crude extract were separated by sodium dodecyl sulfate polyacrylamide gel electrophoresis (SDS-PAGE) under reducing and non-reducing conditions. For the non-reducing condition, the samples were mixed with non-reducing loading buffer (125 mM Tris-Cl, pH 6.8, 20% glycerol, SDS, 0.1% bromophenol blue) and separated on 10% SDS-PAGE. Under the reducing condition, the buffer contained 5% β-mercaptoethanol. For protein detection, the gels were stained with Coomassie brilliant blue (CBB) (Ready to Use; Nacalai Tesque, Kyoto, Japan) following the manufacturer’s instructions. For western blot analysis, the separated proteins in polyacrylamide gel were transferred to a polyvinylidene difluoride (PVDF) membrane (Millipore, Bedford, MA). The membrane was blocked with skim milk (5%) in PBS-T (1.47 mM KH_2_PO_4_, 10 mM Na_2_HPO_4_, 2.7 mM KCl, 137 mM NaCl, 0.05% Tween-20, pH 7.4) for 1 h. The membrane was then washed with PBS-T and incubated with primary antibody anti-human IgG (H+L chain) rabbit IgG (lot WI3374611; Invitrogen) at a dilution of 1:5000, followed by secondary antibody anti-rabbit-HRP IgG (Sigma, St. Louis, Missouri, United States) at a dilution of 1:10,000. Finally, the Varlilumab signals were visualized by adding Immobilon Forte Western HRP substrate (Millipore), and the chemiluminescence was imaged by using an iBright Imaging System (Invitrogen).

### Protein A Sepharose purification

Leaves were harvested and extracted in a cold extraction buffer (2 ml buffer for 1 g of leaves). The plant debris was removed by centrifugation at 15,000 x *g* for 10 min at 4°C. Then the starches and small plant debris were pelleted by adjusting the pH to 7.0 using sodium hydroxide and centrifuged at 15,000 x *g* for 10 min at 4°C. Finally, the clarified extract was passed through filter paper using a vacuum, and the plant-derived antibody was purified by using rProtein A Sepharose fast flow (Pharmacia Biotech AB, Uppsala, Sweden). The column was equilibrated with a 10 column volume (CV) of equilibration buffer (20 mM sodium phosphate, pH 7.0). The plant extract was loaded at a speed of 2 ml/min. The unbound proteins were washed out of the column using 10 CV of wash buffer (20 mM sodium phosphate, pH 7.0). The purified antibody was eluted from the column by using 10 CV of elution buffer (100 mM glycine, 200 mM L-arginine, pH 3.0) and neutralized immediately with neutralizing buffer (1M Tris buffer, pH 8.0).

### Antibody quantification by ELISA

A 96-well plate was coated with 50 μL of goat anti-human IgG (H+L chain) (MBL, Woburn, UK) 1:10000 in 1x PBS buffer and incubated overnight at 4°C. The plate was washed three times with 0.05% Tween-20 in PBS (PBS-T) buffer and blocked with 5% BSA in 1x PBS buffer for 1 h at room temperature (RT). After three additional washings with PBS-T, human plasma IgG (Calbiochem, La Jolla, CA) or plant crude extracts were added into the wells and the plate was incubated at RT for 1 h. The plate was then washed, supplemented with 50 μL of anti-human IgG (H+L chain) conjugated with HRP and incubated for 1 h at RT. The plate was developed using SIGMAFAST™ OPD (Sigma) substrate solution (200 μL/well). The reaction was stopped by addition of 1 M HCl (50 μL/well) and the antibodies were measured at 450 nm with a Bio-Rad imark™ microplate reader.

### *N*-glycan analysis

Purified Varlilumab was separated on SDS-PAGE under reducing conditions and stained with CBB solution. The heavy chain was excised from the gel and destained with 50 mM NH_4_HCO_3_: acetonitrile (1:1 v/v) with overnight intermittent vortex mixing. Then, the heavy chains of Varlilumab were in-gel digested using Trypsin Gold (Promega, Madison, WI) in ProteaseMAX™ Surfactant (Promega) at 50°C for 1 h. The digested peptides were collected from the gel and the reaction was terminated by adding trifluoacetic acid to a final concentration of 0.5%. Digested glycopeptides were applied to a nanoLC-MS/MS system with an ESI-Qq-TOF mass spectrometer (micrOTOF-Q II; Bruker Daltonics, Bremer, Germany) and a nanoLC system (1,200 series; Agilent Technologies, Palo Alto, CA) incorporating a trap column (5 µm, 0.3 x 5 mm) and an analytical column (3.5 µm, 0.075 x 150 mm), both packed with Zorbax 300SB C-18 (Agilent Technologies).

### Antigen-binding assay (CD27 ELISA)

The CD27 ELISA was carried out using recombinant human CD27 (R&D Systems, Minneapolis, MN). Briefly, human CD27 was coated to ELISA plates overnight at 4°C, followed by a blocking with 5% BSA. Each of the following was incubated for 1 h at RT: purified soil-derived Varlilumab, hydroponic-derived Varlilumab, CHO-derived Varlilumab (Thermo Fisher Scientific) as a positive control, and a human plasma IgG (Calbiochem) as a negative control. A goat anti-human IgG (H+L)-HRP (horseradish peroxidase) antibody and substrate SIGMAFAST™ OPD (Sigma) were used for detection. Samples were analyzed at 450 nm using a Bio-Rad imark™ microtiter plate reader.

### Statistical analysis

All experiments were performed in three independent replicates. Statistical evaluations were performed using the Minitab software package. Values of *p*<0.05 were considered to indicate statistical significance.

## Results

### Transient production of Varlilumab in *N. benthamiana*

**Figure 1:**
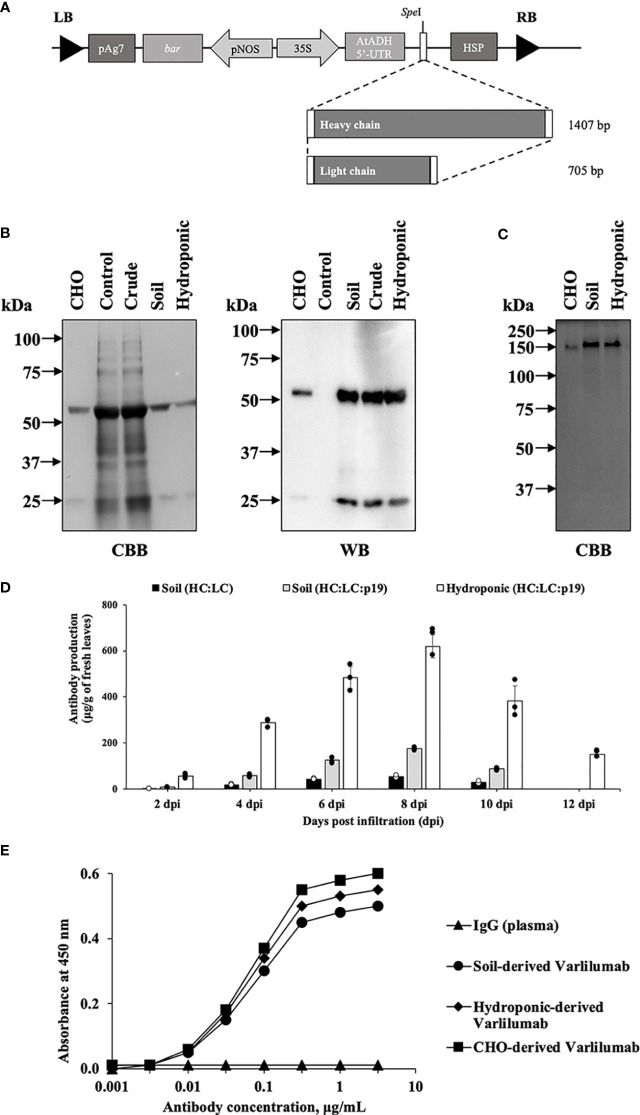
Transient production of Varlilumab in *Nicotiana benthamiana*. **(A)** Schematic representation of plant expression vectors of Varlilumab. CaMV 35S, *Cauliflower mosaic virus* 35S promoter; AtADH 5’-UTR, 5’-untranslated region of *Arabidopsis* alcohol dehydrogenase gene as a translational enhancer; HC, heavy chain Varlilumab; LC, light chain Varlilumab; HSP, *Arabidopsis* heat shock protein terminator; pNOS, Nopaline synthase promoter; *bar*, Bialaphos resistant as a selectable marker gene in plant; pAg7, Agropine synthase gene terminator; and LB and RB, left and right borders of T-DNA, respectively. **(B)** CBB staining and western blotting analysis of Varlilumab. The results under reducing conditions are shown as follows. Lane 1: CHO-derived recombinant human Varlilumab as a positive control. Lane 2: Vector control. Lane 3: Crude extract. Lane 4: Purified Varlilumab derived from soil-based *N. benthamiana.* Lane 5: Purified Varlilumab derived from hydroponic-based *N. benthamiana.* **(C)** CBB staining of Varlilumab. The results under non-reducing conditions are shown as follows. Lane 1: CHO-derived Varlilumab. Lane 2: Purified soil-based Varlilumab. Lane 3: Purified hydroponic-based Varlilumab. **(D)** Leaves were collected after 2, 4, 6, 8, 10 and 12 dpi, and quantified by ELISA. The amount of antibody was shown in micrograms of antibody per gram of fresh weight. Data are means ± SD of samples from three independent infiltration experiments. **(E)** Antigen-binding assay of Varlilumab produced in soil- and hydroponic-based *N. benthamiana*.

To produce recombinant Varlilumab in *N. benthamiana* plants, a modified plant expression vector was used (Sariyatun et al., 2021). The genes encoding the heavy and light chain were individually inserted into the plant expression vector (**Figure 1A**). The plasmids were transferred into *Agrobacterium tumefaciens* LBA4404 using electroporation. Transient production was performed using vacuum infiltration, followed by protein extraction and protein A affinity chromatography purification. The expression and purity of plant-derived Varlilumab were analyzed by SDS-PAGE to confirm the size and integrity of the antibody (**Figures 1B, C**). CBB staining and WB using rabbit anti-human IgG (H+L) and anti-rabbit IgG-horseradish peroxidase (HRP) were used to confirm the production of Varlilumab fragments in *N. benthamiana* and successful purification from the Varlilumab-expressing leaves. As shown in **Figure 1B**, plant-derived Varlilumab was detected at 55 kDa (H) and 26 kDa (L) under reducing conditions. A band corresponding to the assembled antibody was present at approximately 150 kDa under non-reducing conditions, indicating that the antibody was in fully assembled form (H_2_L_2_). There were no differences in size between the purified soil-based and hydroponic-based Varlilumab in CBB staining or WB (**Figure 1C**). These results confirmed that H and L were co-expressed, resulting in the expression of fully assembled Varlilumab in *N. benthamiana*.

Infiltrated leaves were harvested at 2, 4, 6, 8, and 10 days post-infiltration (dpi). Varlilumab was quantified in total protein extracts from each sample by an enzyme-linked immunosorbent assay (ELISA). Transient expression of RNA silencing suppressor p19 was tested to determine its efficiency for increasing the expression of Varlilumab. The highest antibody production was observed at 8 dpi (54 ± 4 µg/g of fresh weight), and co-expression with p19 increased the levels of accumulated antibodies by 3.2-fold (174 ± 5 µg/g of fresh weight) in soil-grown plants (**Figure 1D**). Surprisingly, the expression of Varlilumab in hydroponic-based *N. benthamiana* reached 618 ± 50 µg/gram of fresh weight, 3.5-fold higher than the expression in soil-based *N. benthamiana.* Even at 12 dpi, the hydroponic systems showed high-level Varlilumab production of 151 ± 12 µg/gram of fresh weight, whereas soil-based agroinfiltrated *N. benthamiana* died after 10 dpi. Therefore, co-expression with p19 was necessary and 8 dpi was chosen as the collection day for antibody purification.

We next performed an antigen-binding assay using human CD27 marker (**Figure 1E**). Both soil- and hydroponic-derived Varlilumab showed human CD27-binding affinity similar to that of commercial Chinese hamster ovary (CHO)-derived Varlilumab. A human plasma antibody that cannot bind to the human CD27 protein was used as a negative control. The results indicated that both soil- and hydroponic-based Varlilumab were successfully produced as functional antibodies in *N. benthamiana*.

### *N*-glycan analysis of Varlilumab produced in CHO cells and *N. benthamiana*

**Figure 2:**
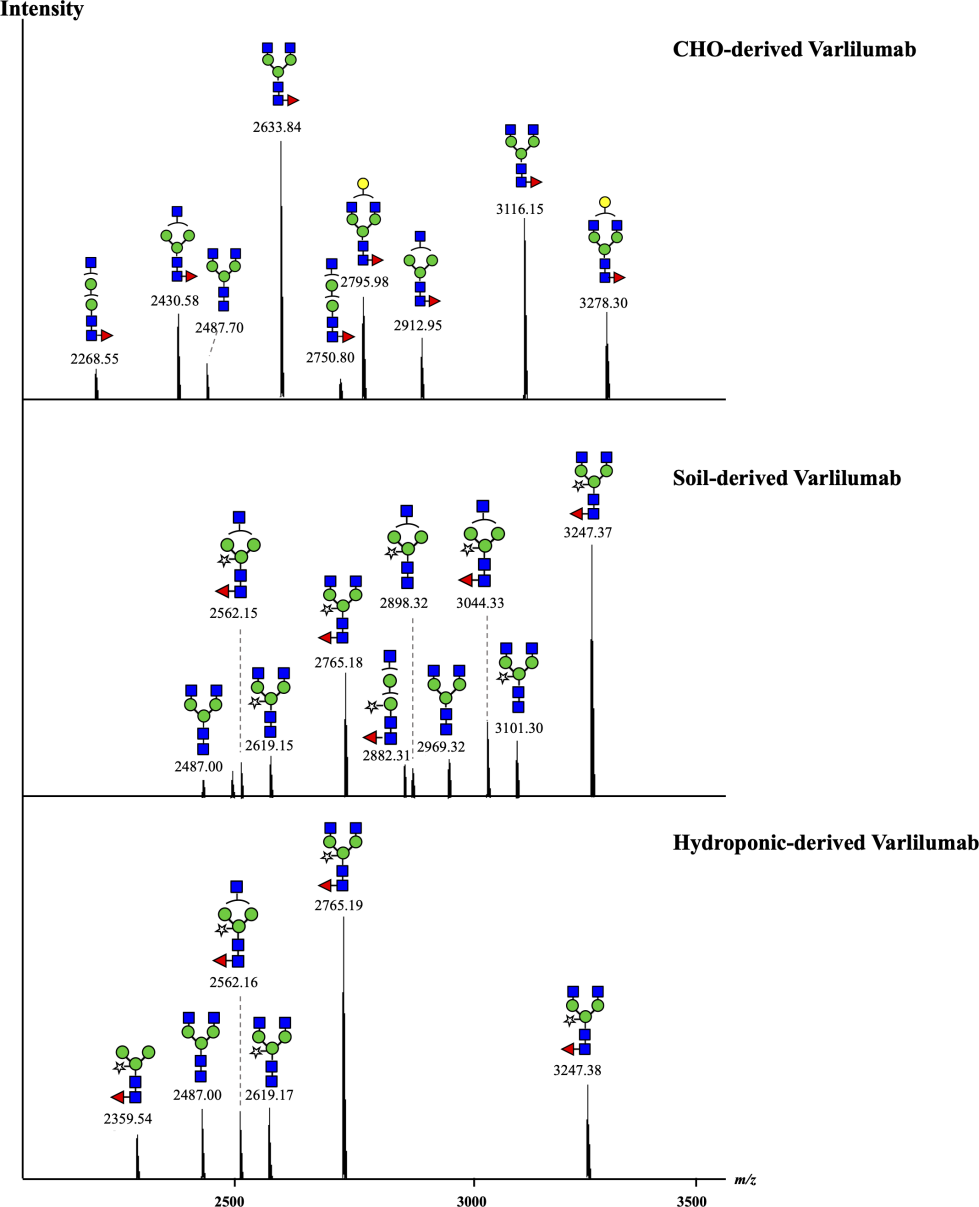
*N*-glycan profile of Varlilumab produced in CHO cells, soil- and hydroponic-based *N. benthamiana.* The deconvoluted MS spectra show the mass [M + H]^+^ of the tryptic glycopeptides carrying an *N*-glycosylation site. The tryptic glycopeptides contained EEQYNSTYR and TKPREEQYNSTYR with a missed cleavage site in the middle due to the steric hindrance of the glycans or the interference of proline near the cleavage site. Both of the glycopeptides were detected in the three analyzed samples.

To investigate the *N*-glycan profiles of Varlilumab produced in CHO cells and *N. benthamiana*, we conducted an *N*-glycan analysis of purified Varlilumab produced in CHO cells, soil-based *N. benthamiana*, and hydroponic-based *N. benthamiana* (**Figure 2**). The trypsin-digested *N*-glycopeptides were analyzed using nanoLC-MS/MS. The percentages of each structure in the total *N*-glycan profile were also determined (**Tables 1**, **2**). In CHO-derived Varlilumab, five *N*-glycoforms were observed. The most abundant *N*-glycopeptide was GN2F, accounting for 51.4% of the total, followed by single-branched β1,4-galactosylated IgG at 21.5% (**Table 1**). As expected, the *N*-glycan analysis of the soil-based and hydroponic-based Varlilumab revealed high levels of β1,2-xylosylated and α1,3-fucosylated *N*-glycan variants, accounting for 72.3% and 76.8% of the total profile, respectively. GN2XF was also a predominant structure, accounting for 54.5% and 58.5% of the profile in soil- and hydroponic-based Varlilumab, respectively (**Table 2**). The Lewis a (Le^a^) structure was not observed in the un-modified setups of either soil- or hydroponic-grown *N. benthamiana* plants.

**Table 1:** Composition of the *N*-glycan structures in CHO-derived Varlilumab.

Abbreviation	Structure	*N*-glycopeptide ratio (%)
GNM2F	GlcNAcMan2Fuc	5.8
GNF	GlcNAcMan3Fuc	17.1
GN2	GlcNAc2Man3	4.2
GN2F	GlcNAc2Man3Fuc	51.4
Gal(β1,4)GN2F	GalGlcNAc2Man3Fuc	21.5
	**Total**	**100**

**Table 2:** Composition of the *N*-glycan structures in Varlilumab produced in *N. benthamiana* soil and hydroponic systems.

Abbreviation	Structure	Soil-based (%)	Hydroponic-based (%)
M3XF	Man3XylFuc	–	7.3
GNM2XF	GlcNAcMan2XylFuc	4.5	–
GNX	GlcNAcMan3Xyl	4.0	–
GN2	GlcNAc2Man3	8.9	11.6
GNXF	GlcNAcMan3XylFuc	14.7	11.0
GN2X	GlcNAc2Man3Xyl	13.5	11.6
GN2XF	GlcNAc2Man3XylFuc	54.5	58.5
	**Total**	**100**	**100**
**β1,2-Xylosylated** **β1,2-Xylosylated and α1,3-Fucosylated** **Lewis a structure**		17.5 72.3 -	11.6 76.8 -

### Co-expression of Varlilumab with β1,4-galactosyltransferase or β1,3-galactosyltransferase in *N. benthamiana*

**Figure 3:**
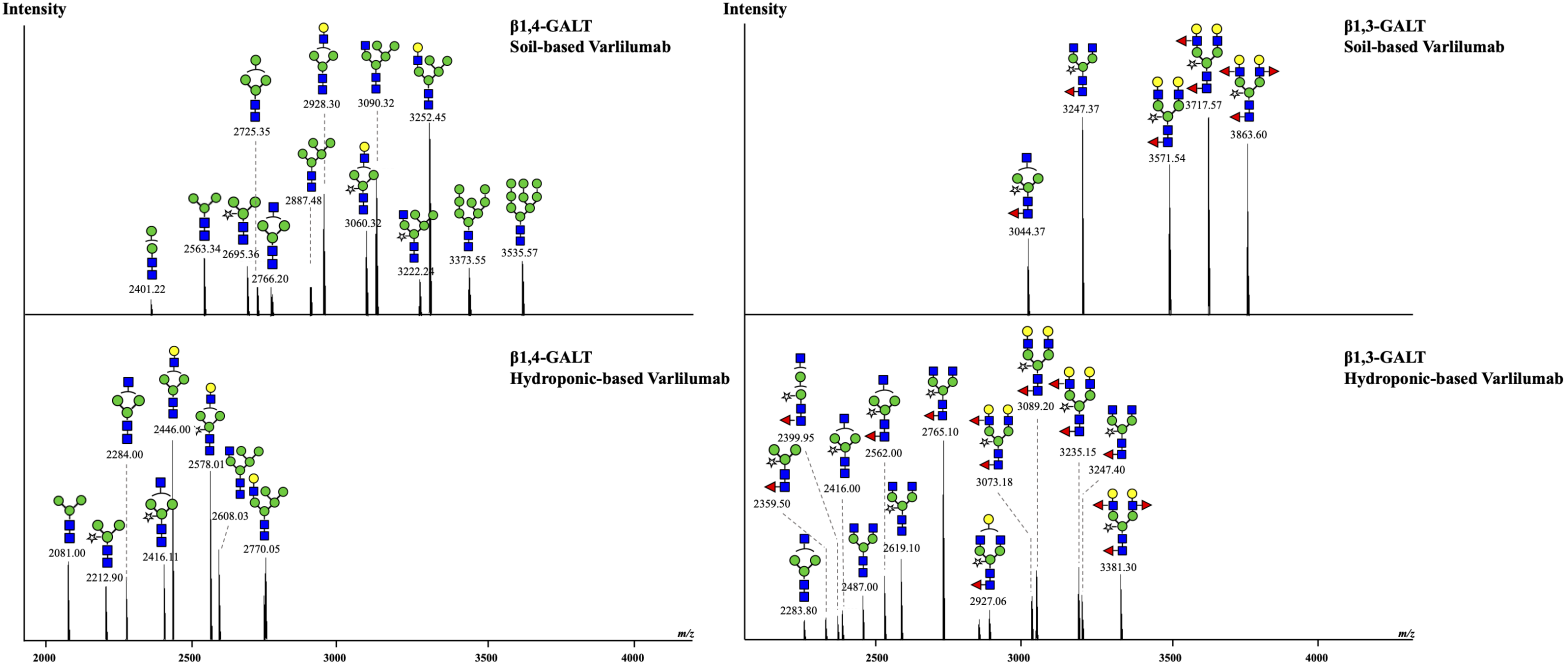
Co-expression of Varlilumab with β1,4-galactosyltransferase (β1,4-GALT) or β1,3-galactosyltransferase (β1,3-GALT) in *N. benthamiana* harvested at 8 dpi. Mass spectra of glycopeptides derived from co-expression of Varlilumab with β1,4-GALT or β1,3-GALT produced in soil- and hydroponic-based *N. benthamiana* plants. In this analysis, the trypsin-digested glycopeptides from soil-based Varlilumab, contained TKPREEQYNSTYR while these from hydroponic-based Varlilumab carried EEQYNSTYR.

To improve the effectiveness of plant-derived Varlilumab, *N*-glycan engineering was performed with the aim of producing Varlilumab with Gal residues on its *N*-glycan. Previous research indicated that Gal residues stabilize the antibody’s configuration, improving the binding between the Fc region and Fc receptors (Kiyoshi et al., 2018). In the previous *N*-glycan analysis, single-branched β1,4-galactosylated IgG accounted for 21.5% of CHO-derived Varlilumab, and in un-modified plants, more than half of plant-derived Varlilumab bore a GNGN core structure, which could be extended via β1,4-linked galactose by β1,4-GALT (Bohlender et al., 2022). In this experiment, both soil- and hydroponic-based *N. benthamiana* plants were used for the co-expression of Varlilumab with murine β1,4-GALT. The constituent *N*-glycans of each modification in Varlilumab were determined by *de novo* sequencing of the trypsin-digested *N*-glycopeptides using nanoLC-MS/MS analysis. The *N*-glycopeptides bearing the sequence of N297 were successfully detected. The majority of the *N*-glycans of Varlilumab co-expressed with β1,4-GALT were galactose-terminated, with the galactose-terminated *N*-glycans accounting for 42.5% and 55.3% of total *N*-glycan variants in soil-based and hydroponic-based plants, respectively (**Figure 3** and **Table 3**). However, the modified product formed by β1,4-GALT co-expression was a hybrid structure having a single Galβ1,2-1,4-GlcNAc (42.5% of total *N*-glycan variants in soil-based and 55.3% of that in hydroponic-based plants). Moreover, a non-mature ER-specific glycans (Man9) and high mannose structures were also observed in soil-based plants. Transient expression of β1,4-GALT in *N. benthamiana* results in a partial galactosylation in plant-derived Varlilumab.

**Table 3:** Composition of the *N*-glycan structures in Varlilumab co-expressed with β1,4-GALT *in N. benthamiana*.

Abbreviation	Structure	Soil-based (%)	Hydroponic-based (%)
M2	Man2	1.7	–
M3	Man3	8.2	9.6
M4	Man4	5.4	–
M5	Man5	5.3	–
M8	Man8	5.1	–
M9	Man9	5.7	–
M3X	Man3Xyl	5.2	6.9
GNM3	GlcNAcMan3	2.3	7.7
GNM3X	GlcNAcMan3Xyl	–	9.4
GNM5	GlcNAcMan5	14.8	11.1
GNM5X	GlcNAcMan5Xyl	3.7	–
Gal(β1,4)GNM3	GalGlcNAcMan3	12.5	24.8
Gal(β1,4)GNM3X	GalGlcNAcMan3Xyl	8.8	20.5
Gal(β1,4)GNM5	GalGlcNAcMan5	21.2	10.0
	**Total**	**100**	**100**
**Terminal β1,4-Gal residue** **β1,2-Xylosylated** **Oligomannosidic structure**		42.5 17.7 21.5	55.3 36.8 -

To target mammalian β1,4‐GALT to the *trans* or late Golgi of plants, the native localization domain of mammalian β1,4‐GALT was replaced by the *N*-terminal CTS of *Arabidopsis thaliana* β1,2-XYLT (Saint-Jore-Dupas et al., 2006) or rat α2,6-ST (Strasser et al., 2009). In this study, we chose β1,3-GALT because its subcellular localization was predicted to be exclusively located in the plant Golgi apparatus. *A. thaliana* β1,3-GALT was co-expressed with Varlilumab to observe how it modifies the *N*-glycan profiles of the antibody. The *N*-glycan analysis indicated that the formation of the Le^a^ structure was enhanced (**Figure 3** and **Table 4**). In previous analysis, there was no Le^a^ structures observed in the un-modified setups of both soil- and hydroponic-based *N. benthamiana* plants (**Table 2**). In this modification, there were 46.1% and 22.7% of Le^a^ structures formed in the soil- and hydroponic-based plants, respectively. In contrast, the percentage of the substrate acceptor of β1,3-GALT, i.e., GN2XF decreased from 54.5% to 25.3% in the soil-based plants and from 58.5% to 27.3% in the hydroponic-based plants. Furthermore, *N*-glycan structures containing both β1,2-Xyl and α1,3-Fuc residues were different between the soil- and hydroponic-based *N. benthamiana* plants. In the soil-based plants, the combined β1,2-XYLT and ɑ1,3-fucosyltransferase (FUCT) worked cooperatively to form 100% of *N*-glycans contained both β1,2-Xyl and α1,3-Fuc residues whereas in hydroponic plants, 13.7% of *N*-glycan structures contained only β1,2-Xyl residues. In short, co-expression with *A. thaliana* β1,3-GALT improved galactosylation efficiency and enhanced the formation of the Le^a^ structure of plant-derived Varlilumab compared to the un-modified setup.

**Table 4:** Composition of the *N*-glycan structures in Varlilumab co-expressed with β1,3-GALT *in N. benthamiana*.

Abbreviation	Structure	Soil-based (%)	Hydroponic-based (%)
M3XF	Man3XylFuc	–	2.8
GNM3	GlcNAcMan3	–	2.5
GNX	GlcNAcMan3Xyl	–	3.7
GNXF	GlcNAcMan3XylFuc	9.8	8.0
GN2M2XF	GlcNAc2Man2XylFuc	–	3.0
GN2	GlcNAc2Man3	–	5.4
GN2X	GlcNAc2Man3Xyl	–	10.0
GN2XF	GlcNAc2Man3XylFuc	25.3	27.3
GalGN2XF	GalGlcNAc2Man3XylFuc	–	3.8
Gal(F)GN2XF	GalFucGlcNAc2Man3XylFuc	–	5.6
Gal2GN2XF	Gal2GlcNAc2Man3XylFuc	18.8	10.8
Gal2(F)GN2XF	Gal2FucGlcNAc2Man3XylFuc	24.7	9.0
Gal2F2GN2XF	Gal2Fuc2GlcNAc2Man3XylFuc	21.4	8.1
**Total**		**100**	**100**
**β1,2-Xylosylated** **β1,2-Xylosylated and α1,3-Fucosylated** **Lewis a structure**		- 100 64.9	13.7 78.4 22.7

### Co-expression of Varlilumab with a chimeric protein β1,3β1,4-GALT

**Figure 4:**
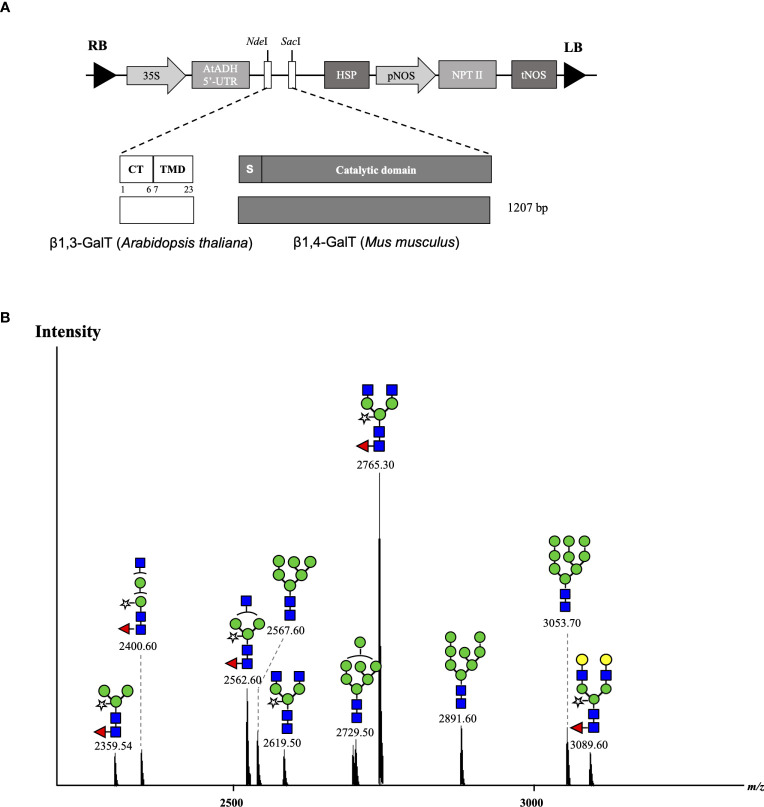
Co-expression of Varlilumab with chimeric β1,3β1,4-GALT in *N. benthamiana* harvested at 8 dpi. **(A)** Schematic representation of plant expression vectors of chimeric β1,3β1,4-GALT. CaMV 35S, *Cauliflower mosaic virus* 35S promoter; AtADH 5’-UTR, 5’-untranslated region of the *Arabidopsis* alcohol dehydrogenase gene as a translational enhancer; CT, Cytoplasmic tail; TMD, Transmembrane domain; HSP, *Arabidopsis* heat shock protein terminator; pNOS, Nopaline synthase promoter; NPT II, Neomycin phosphotransferase gene as a selectable marker gene in plant; tNOS, Nopaline synthase terminator; and LB and RB: left and right borders of T-DNA, respectively. **(B)** Mass spectra of glycopeptides (EEQYNSTYR) derived from co-expression with chimeric β1,3β1,4-GALT produced in soil-based *N. benthamiana* plants.

In our previous experiments, it was suggested that the original CTS region of murine β1,4-GALT acted early in the Golgi network of transiently agroinfiltrated *N. benthamiana* wild type leaves and single β1,4-galactosylated *N*-glycan structures were produced, whereas galactosylation efficiency was improved by co-expression with *A. thaliana* β1,3-GALT. Therefore, we hypothesized that the cytoplasmic tail (CT) and transmembrane domain (TMD) of β1,3-GALT would result in hybrid-type *N*-glycans in the antibody. Previous studies have also demonstrated that the *N*-terminal CTS domain of β1,4-GALT is important in determining the *N*-glycosylation profile of recombinant proteins in plants. When the original CTS of β1,4-GALT was replaced with a rat α2,6-sialytransferase CTS domain, more homogeneous biantennary galactosylated *N*-glycan profiles were achieved, as this swapping led to translocation of GALT to the *trans-*Golgi compartments (Schoberer et al., 2009; Castilho et al., 2011). In this study, to investigate whether the expression of CT and TMD of β1,3-GALT results in hybrid-type *N*-glycans in the antibody, the original CT and TMD regions of mouse β1,4-GALT were replaced by the first 23 amino acids encoding for CT and TMD of β1,3-GALT. The chimeric β1,3β1,4-GALT was placed in the pRI201AN vector (**Figure 4A**). This construct was co-expressed with Varlilumab and the *N*-glycans profile of modified Varlilumab was analyzed using nanoLC-MS/MS. Biantennary galactosylated *N*-glycan (*m/z* 3089.6) was observed (**Figure 4B**) and accounted for 4.4% of the total *N*-glycan profile. Moreover, non-mature ER-specific glycans (Man9) accounted for 7.3% of the total profile, high mannose structures (Man6, Man7 and Man8) made up 20.8%, and the remaining 67% are complex *N*-glycans carrying both β1,2-Xyl and α1,3-Fuc residues (**Table 5**). This result suggested that CT and TMD of *A. thaliana* β1,3-GALT are sufficient to form biantennary β1,4-galactosylated *N*-glycans in plant-derived Varlilumab.

**Table 5:** Composition of the *N*-glycan structures in Varlilumab co-expressed with chimeric β1,3β1,4-GALT *in N. benthamiana*.

Abbreviation	Structure	*N*-glycopeptide ratio (%)
M3XF	Man3XylFuc	4.1
GNM2XF	GlcNAcMan2XylFuc	4.6
GNXF	GlcNAcMan3XylFuc	12.4
GN2X	GlcNAc2Man3Xyl	4.8
GN2XF	GlcNAc2Man3XylFuc	41.5
Gal2(β1,4)GN2XF	Gal2GlcNAc2Man3XylFuc	4.4
M6	Man6	7.2
M7	Man7	5.8
M8	Man8	7.8
M9	Man9	7.3
	**Total**	100
**Terminal β1,4-Gal residue** **β1,2-Xylosylated and α1,3-Fucosylated** **Oligomannosidic structure** **Lewis a structure** **β1,2-Xylosylated**		4.4 67.0 20.8 - 4.8

## Discussion

Cancer immunotherapy has become an attractive treatment option. In particular, the use of monoclonal antibodies—including Varliumab (anti-human CD27)—in cancer treatment has been studied in multiple clinical trials, but the anti-CD27 mAb has been produced only in CHO cells, and contamination and high cost are concerns when using CHO cells (Nosaki et al., 2021a). Therefore, we developed an alternative platform for producing an effective Varlilumab in plants. Transgenic plants are still prohibited in many countries, and the generation of stable plant transformants is time-consuming (Rivera et al., 2012; Sethi et al., 2021). To tackle these obstacles, transient expression technology was used in this study. The recombinant Varlilumab was expressed in *N. benthamiana* leaves within 8 days of infiltration and successfully assembled. With co-expression of p19, an RNA silencing suppressor, mAb was accumulated at an average of 174 µg/g of fresh leaf weight. This production was higher than that of nivolumab (140 µg/g of fresh leaf weight) in a transient production in *N. benthamiana* using geminiviral vectors (Rattanapisit et al., 2019). The result showed that the purified plant-derived Varlilumab in both soil- and hydroponic-based systems assembled correctly into a tetramer and retained *in vitro* efficacy similar to those of commercial mammalian cell-produced Varlilumab.

Of note, a high expression of Varlilumab was observed in hydroponic systems. Varlilumab expression reached 618 µg/g of fresh leaf weight in hydroponic *N. benthamiana*, which was 3.5 times greater than the expression achieved in soil-based *N. benthamiana*. The antibody production was rapidly increased from 4 dpi (288 µg/g of fresh leaf weight) and stayed high until 12 dpi (151 µg/g of fresh leaf weight). Hydroponic cultivating conditions have been shown to increase the accumulation of ascorbic acid in plants (Buchanan and Omaye, 2013). Ascorbic acid has been shown to suppress necrosis caused by transient expression in *N. benthamiana* (Nosaki et al., 2021b) and to scavenge excess reactive oxygen species (ROS) generated by *A. tumefaciens* (Wojtaszek, 1997). Therefore, an accumulation of ascorbate acid in hydroponic-based *N. benthamiana* plants might contribute to an improvement of transformation, enhancement of agroinfiltration and maintenance of the health of agroinfiltrated plants. To our knowledge, this is the first study to produce recombinant Varlilumab in a plant-based platform. Our results indicate that this system has the potential to be competitive for the recombinant production of antibodies with high yield.

**Figure 5:**
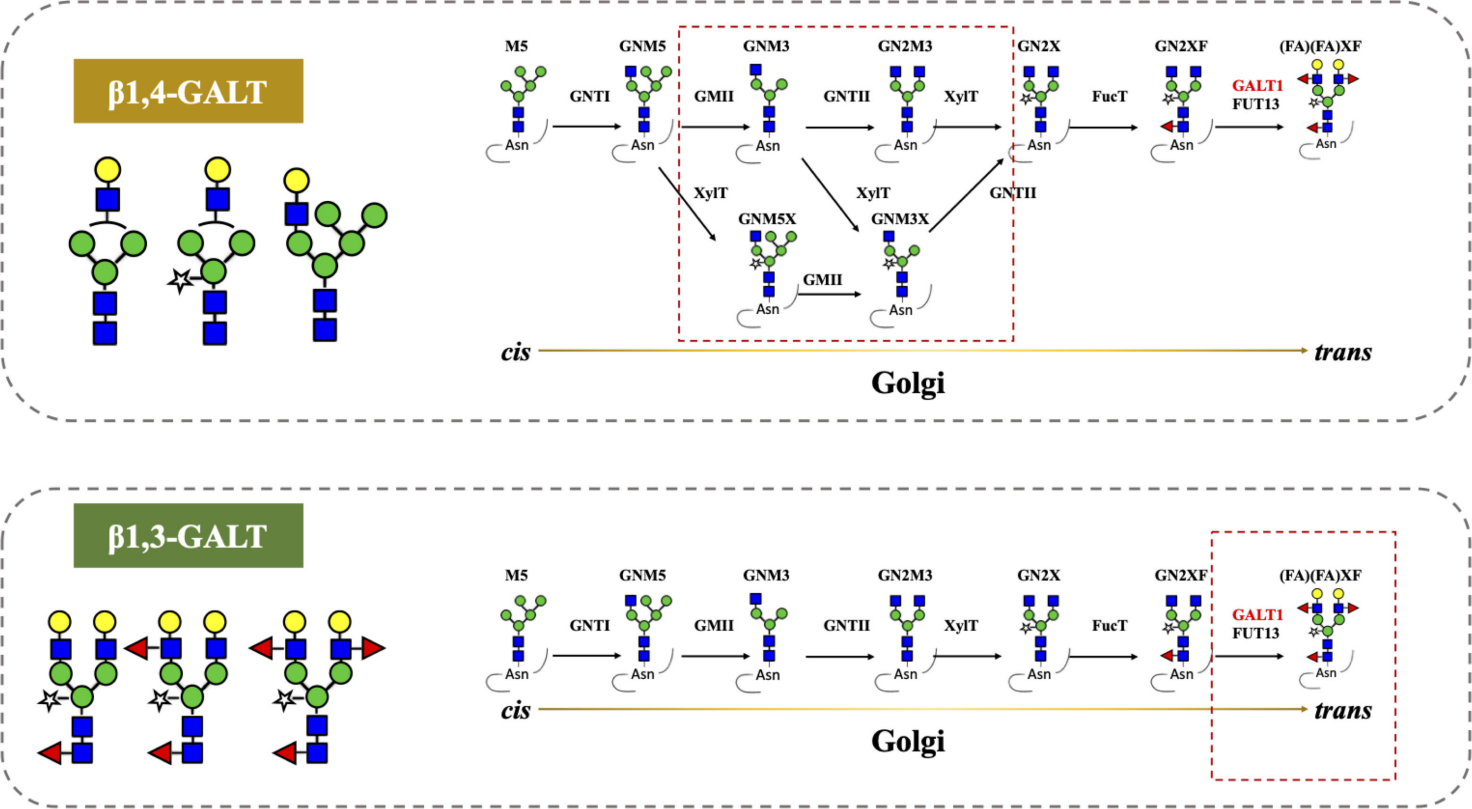
Co-expression of β1,4-galactosyltransferase (β1,4-GALT) and β1,3-galactosyltransferase (β1,3-GALT) in *N. benthamiana.* GnTI, *N*-Acetylglucosaminyltransferase I; GMII, Golgi-α-mannosidase II; GnTII, *N*-Acetylglucosaminyltransferase II; XylT, β1,2-Xylosyltransferase; FucT, core α1,3-Fucosyltransferase; GALT1, β1,3-Galactosyltransferase; FUT13, α1,4-Fucosyltransferase. M5, Man5; GNM5, GlcNAcMan5; GNM3, GlcNAcMan3; GN2M3, GlcNAc2Man3; GN2X, GlcNAc2Man3Xyl; GN2XF, GlcNAc2Man3XylFuc; (FA)(FA)XF, Gal2Fuc2GlcNAc2Man3XylFuc.

Previous reports described that the antibodies produced in tobacco do not have β1,4-Gal terminals, which show better performance in their effector functions, as plants lack mammalian β1,4-GALT. Previous studies reported the stable expression of human β1,4-GALT in tobacco cell-lines and transgenic tobacco plants (Palacpac et al., 1999; Bakker et al., 2001). Although these approaches achieved galactosylated IgGs, the hurdles are laborious and time-intensive. Therefore, transient expression of Varlilumab and co-expression of GALT were selected to obtain galactosylated Varlilumab produced in *N. benthamiana* in a short period of time. Despite the fact that transient expression of β1,4-GALT in tobacco plants results in galactosylation of Varlilumab, the *N*-glycan modification was partially processed. The GalGNM5 structure was found and β1,2-Xyl residue was also present in β1,4-Gal-containing *N*-glycans, suggesting that β1,4-GALT blocked the function of Golgi-α-mannosidase II and acted early in the Golgi apparatus together with β1,2-xylosyltransferase (XYLT). Moreover, the presence of xylose-bearing *N*-glycan structures (GNM5X or GNM3X) in soil-based Varlilumab (17.7%) and hydroponic-based Varlilumab (36.8%) indicated that β1,2-XYLT worked actively in the *medial*-Golgi (**Figure 5**). On the contrary, Varlilumab with β1,4-GALT co-expressed contained glycans with no detectable α1,3-fucose. This may suggest that the introduced β1,4-GALT is integrated in the Golgi apparatus at a location which is adjacent to the α1,3-FUCT. Thus, the two glycosyltransferases are competing for the same substrates passing by. This was previously observed with the stable expression of human β1,4-GALT in transgenic tobacco cell lines and transgenic plants of *N. tabacum* (Palacpac et al., 1999; Bakker et al., 2001; Bakker et al., 2006). Moreover, the presence of 5.7% of a non-mature ER-specific glycans (Man9) and 21.5% of high-mannose-type glycans could be associated with a fraction of proteins “en-route”, as reported previously for other plant-derived antibodies produced by transient expression in soil-based *N. benthamiana* (Sriraman et al., 2004). However, hydroponic-derived Varlilumab showed a dissimilar *N*-glycan profile to Varlilumab produced in soil-based *N. benthamiana*, indicating that the hydroponic cultivation would alter *N*-glycosylation patterns. Although galactosylated Varlilumab was found in this co-expression with β1,4-GALT, biantennary *N*-glycans with β1,4-Gal were not achieved. To improve galactosylation by β1,4-GALT in plants, β1,4-GALT has been coupled with different membrane anchorage domains of tobacco, such as GNTI (Vézina et al., 2009), XYLT (Saint-Jore-Dupas et al., 2006), and ST (Strasser et al., 2009; Forthal et al., 2010; Castilho et al., 2011; Loos et al., 2015; Schneider et al., 2015; Kallolimath et al., 2018).

In this study, we also investigated *A. thaliana* β1,3-GALT, the subcellular localization of which was predicted to lie exclusively in the Golgi apparatus (Strasser et al., 2007). In parallel, we co-expressed β1,3-GALT with Varlilumab in both soil- and hydroponic-grown *N. benthamiana* plants. The *N*-glycan analysis of Varlilumab revealed successful modification in both soil- and hydroponic-based *N. benthamiana* plants when compared to the un-modified setup (**Figure 3** and **Table 4**). Le^a^ structures were found in the soil- and hydroponic-based plants at rates of 64.9% and 22.7%, respectively. Le^a^ [Fucα1-4(Galβ1-3)GlcNAc-R] structures require the continuous extension of β1,3-galactose and α1,4-fucose residues by β1,3-GALT and α1,4-fucosyltransferase (FUT13) in the *trans*-Golgi compartments (Strasser et al., 2007). In soil-based *N. benthamiana*, all of the *N*-glycan structures of Varlilumab carried both β1,2-Xyl and α1,3-Fuc residues and 64.9% of Le^a^ structures were formed, suggesting that Varlilumab produced in the soil-based plants properly passed through the *trans*-Golgi network. By contrast, Varlilumab produced in a hydroponic system had a smaller amount of Le^a^ structures (22.7%), and 13.7% of the *N*-glycan variants carried only the β1,2-Xyl residue, indicating these amounts of Varlilumab being en-routed in hydroponic-based *N. benthamiana* plants (Sriraman et al., 2004).

The transient expression of mammalian GALT in *N. benthamiana* results in galactosylation of antibodies and reflects a dynamic state of the *N*-glycosylation process with the presence of non-mature ER-specific glycans and fucose-carrying glycan structures. β1,4-GALT localizes correctly but early in the plant Golgi apparatus, while the endogenous β1,3-GALT acts in the *trans*-Golgi compartments (**Figure 5**). Thus, a chimeric of the mammalian β1,4-GALT catalytic domain and transmembrane domain of β1,3-GALT was generated to improve the galactosylation of β1,4-GALT in *N. benthamiana*. Specifically, the first 23 aa encoding for CT and TMD of β1,3-GALT fused with the stem, and the catalytic domain of β1,4-GALT (**Figure 4A**) was co-expressed with Varlilumab. *N*-glycan analysis of the modified Varlilumab indicated that 4.4% of the total Varlilumab had biantennary β1,4-galactosylated *N*-glycans (*m/z* 3089.6) (**Figure 4B** and **Table 5**). This finding revealed that CT and TMD of *A. thaliana* β1,3-GALT are sufficient to form biantennary β1,4-galactosylated *N*-glycans in plant-derived Varlilumab.

## Conclusion

In this study, we established a plant-based platform to rapidly produce Varlilumab. Our data demonstrated that Varlilumab can be transiently produced in *N. benthamiana* with a large amount of mAb in a hydroponic system. We also found that co-expression with chimeric β1,4-GALT successfully achieved biantennary β1,4-galactosylated Varlilumab. Gal is important for the effector functions of antibodies, and transient expression significantly reduces the time and material required as compared with the generation of transgenic plants stably expressing antibodies, so this is a major improvement in the *in planta* engineering of antibodies and a critical step toward obtaining recombinant therapeutic *N*-glycoproteins with fully humanized *N*-glycans.

## Data availability statement

The original contributions presented in the study are included in the article/**Supplementary Material**. Further inquiries can be directed to the corresponding author.

## Author contributions

KN performed the experiments and wrote a manuscript. HK assisted with nanoLC-MS/MS analysis and RM helped to verify DNA sequences of Varlilumab. RK and TY established the growing conditions for hydroponic-based *N. benthaminana*. KF supervised the findings of this work. All authors contributed to the article and approved the submitted version.
