# Supplementary material for: Production and N-glycan engineering of Varlilumab in Nicotiana benthamiana
Source: Front Plant Sci. 2023 Aug 8;14:1215580. doi: 10.3389/fpls.2023.1215580 (PMC10442953; doi:10.3389/fpls.2023.1215580)
Supplement: Supplementary file 1 [file DataSheet_1.pdf]

Supplementary Material

Production and *N*-glycan engineering of Varlilumab in *Nicotiana benthamiana*

Nguyen Kim Dua[1], Kajiura Hiroyuki[1,2], Kamiya Ryo[4], Yoshida Takahiro[4], Misaki Ryo[1,2], Fujiyama Kazuhito[1,2, 3*]

[1]International Center for Biotechnology, Osaka University, Osaka, Japan

[2]Industrial Biotechnology Initiative Division, Institute for Open and Transdisciplinary Research Initiatives (OTRI), Osaka University, Osaka, Japan.

[3]Osaka University Cooperative Research Station in Southeast Asia, (OU:CRS), Faculty of Science, Mahidol University, Bangkok, Thailand.

[4]GreenLand-Kidaya group Co Ltd., Fukui, Japan

*** Correspondence:** Fujiyama Kazuhito
fujiyama@icb.osaka-u.ac.jp

Supplementary Table 1. Quantification of *N*-glycan structures of purified Varlilumab produced in CHO cells (MS mode)

**Abbreviation**	**Structure**	**Mass-to-charge ratio (*m/z*)**			
		**EEQYNSTYR**		**TKPREEQYNSTYR**	
		**Theoretical**	**Observed**	**Theoretical**	**Observed**
Peptide + GNM2F	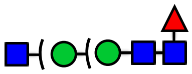	2268.58	2268.55	2750.90	2750.80
Peptide + GNF	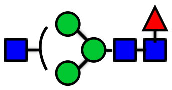	2430.63	2430.58	2913.00	2912.95
Peptide + GN2	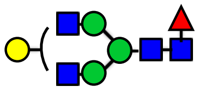	2487.66	2487.70	-	-
Peptide + GN2F	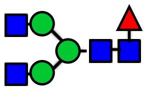	2633.71	2633.84	3116.29	3116.15
Peptide + GalGN2F	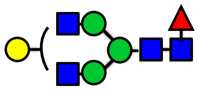	2795.77	2795.98-	3278.34	3278.30

Supplementary Table 2. Quantification of *N*-glycan structures of purified Varlilumab produced in soil-based *N. benthamiana* WT (MS mode)

**Abbreviation**	**Structure**	**Mass-to-charge ratio (*m/z*)**			
		**EEQYNSTYR**		**TKPREEQYNSTYR**	
		**Theoretical**	**Observed**	**Theoretical**	**Observed**
Peptide + GNM2XF	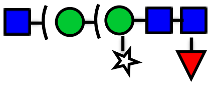	-	-	2882.23	2882.31
Peptide + GNX	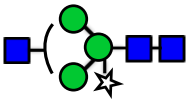	-	-	2898.22	2898.32
Peptide + GN2	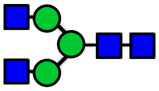	2486.97	2487.00	2969.26	2969.32
Peptide + GNXF	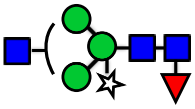	2562.00	2562.15	3044.28	3044.33
Peptide + GN2X	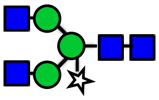	2619.01	2619.15	3101.30	3101.30
Peptide + GN2XF	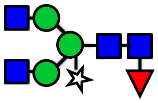	2765.07	2765.18	3247.36	3247.37

Supplementary Table 3. Quantification of *N*-glycan structures of purified Varlilumab produced in hydroponic-based *N. benthamiana* WT (MS mode)

Abbreviation	Structure	Mass-to-charge ratio (*m/z*)			
		EEQYNSTYR		TKPREEQYNSTYR	
		Theoretical	Observed	Theoretical	Observed
Peptide + M3XF	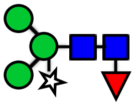	2359.60	2359.54	-	-
Peptide + GN2	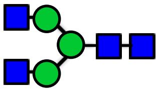	2486.97	2487.00	-	-
Peptide + GNXF	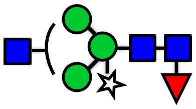	2562.00	2562.16	-	-
Peptide + GN2X	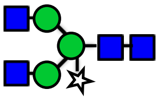	2619.01	2619.17	-	-
Peptide + GN2XF	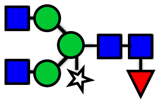	2765.07	2765.19	3247.36	3247.38

Supplementary Table 4. Quantification of *N*-glycan structures of purified Varlilumab produced in *N. benthamiana* WT co-expressed with murine β1,4 galactosyltransferase (MS mode)

Abbreviation	Structure	Mass-to-charge ratio (*m/z*)			
		EEQYNSTYR		TKPREEQYNSTYR	
		Theoretical	Observed	Theoretical	Observed
Peptide + M2	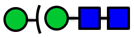	-	-	2401.05	2401.22
Peptide + M3	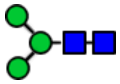	-	-	2563.10	2563.34
Peptide + M4	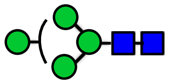	-	-	2725.16	2725.35
Peptide + M5	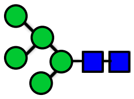	-	-	2887.21	2887.48
Peptide + M8	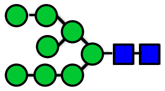	-	-	3373.37	3373.55
Peptide + M9	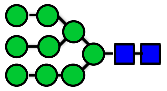	-	-	3535.42	3535.57
Peptide + M3X	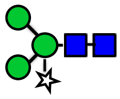	-	-	2695.14	2695.36
Peptide + GNM3	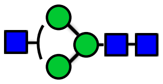	-	-	2766.18	2766.20

Supplementary Table 5. Quantification of *N*-glycan structures of purified Varlilumab produced in hydroponic-based *N. benthamiana* WT co-expressed with murine β1,4 galactosyltransferase (MS mode)

Abbreviation	Structure	Mass-to-charge ratio (*m/z*)			
		EEQYNSTYR		TKPREEQYNSTYR	
		Theoretical	Observed	Theoretical	Observed
Peptide + M3	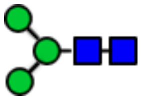	2080.95	2081.00	-	-
Peptide + M3X	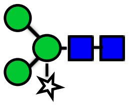	2212.85	2212.90	-	-
Peptide + GNM3	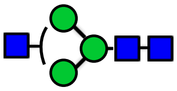	2283.89	2284.00	-	-
Peptide + GNM5	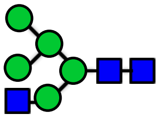	2608.00	2608.03	-	-
Peptide + GNM3X	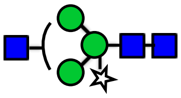	2416.10	2416.11	-	-
Peptide + GalGNM3	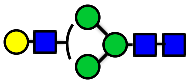	2445.98	2446.00	-	-
Peptide + GalGNM3X	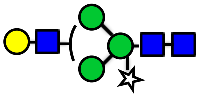	2678.00	2578.01	-	-
Peptide + GalGNM5	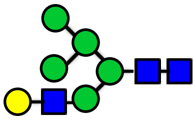	2770.05	2770.05	-	-

Supplementary Table 6. Quantification of *N*-glycan structures of purified Varlilumab produced in soil-based *N. benthamiana* WT co-expressed with *Arabidopsis thaliana* β1,3 galactosyltransferase (MS mode)

Abbreviation	Structure	Mass-to-charge ratio (*m/z*)			
		EEQYNSTYR		TKPREEQYNSTYR	
		Theoretical	Observed	Theoretical	Observed
Peptide + GnXF	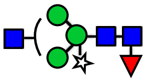	-	-	3044.27	3044.37
Peptide + GNXF	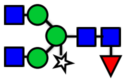	-	-	3247.37	3247.37
Peptide + Gal2GN2XF	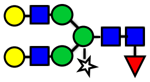	-	-	3571.47	3571.54
Peptide + Gal2(F)GN2XF	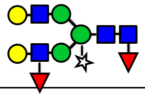	-	-	3717.53	3717.57
Peptide + Gal2F2GN2XF	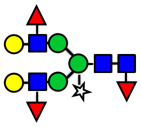	-	-	3863.59	3863.60

Supplementary Table 7. Quantification of *N*-glycan structures of purified Varlilumab produced in hydroponic-based *N. benthamiana* WT co-expressed with *Arabidopsis thaliana* β1,3 galactosyltransferase (MS mode)

**Abbreviation**	**Structure**	**Mass-to-charge ratio (*m/z*)**			
		**EEQYNSTYR**		**TKPREEQYNSTYR**	
		**Theoretical**	**Observed**	**Theoretical**	**Observed**
Peptide + M3XF	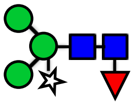	2359.60	2359.50		-
Peptide + GNM3	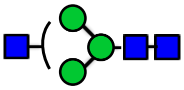	2283.89	2283.80	-	-
Peptide + GNX	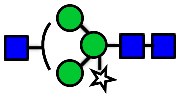	2416.10	2416.00	-	-
Peptide + GNXF	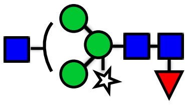	2562.00	2562.00	-	-
Peptide + GNM2XF	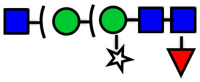	2400.00	2399.95	-	-
Peptide + GN2	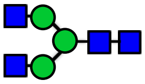	2486.97	2487.00	-	-
Peptide + GN2X	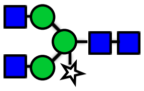	2619.01	2619.10	-	-
Peptide + GN2XF	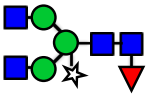	2765.07	2765.10	3247.37	3247.40
Peptide + GalGN2XF	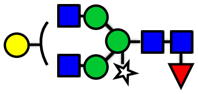	2927.10	2927.06	-	-
Peptide + Gal2GN2XF	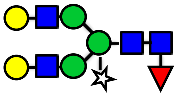	3089.30	3089.20	-	-

Peptide + Gal2(F)GN2XF	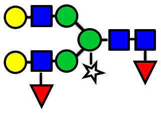	3235.20	3235.15	-	-
Peptide + Gal2F2GN2XF	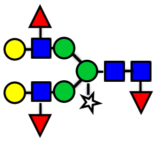	3381.50	3381.30	-	-

Supplementary Table 8. Quantification of *N*-glycan structures of purified Varlilumab produced in soil-based *N. benthamiana* WT co-expressed with chimeric **β1,3β1,4-GALT** (MS mode)

Abbreviation	Structure	Mass-to-charge ratio (*m/z*)			
		EEQYNSTYR		TKPREEQYNSTYR	
		Theoretical	Observed	Theoretical	Observed
Peptide + M3XF	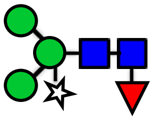	2359.60	2359.54	-	-
Peptide + GNM2XF	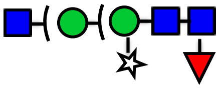	2400.62	2400.60	-	-
Peptide + GNXF	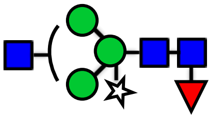	2562.68	2562.60	-	-
Peptide + GN2X	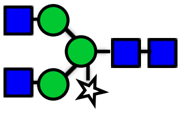	2619.70	2619.50	-	-
Peptide + GN2XF	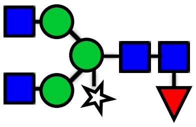	2765.36	2765.30	-	-
Peptide + Gal2GN2XF	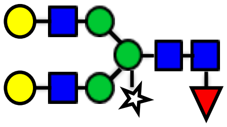	3089.80	3089.60	-	-
Peptide + Man6	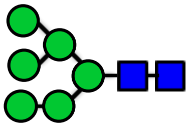	2567.66	2567.60	-	-
Peptide + Man7	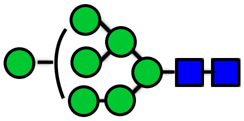	2719.70	2729.50	-	-
Peptide + Man8	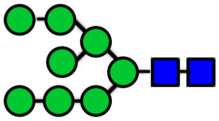	2891.70	2891.60	-	-

Peptide + Man9	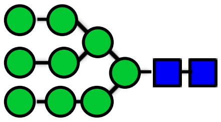	3053.80	3053.70	-	-
